# Cognitive Engagement Mediates the Relationship between Positive Life Events and Positive Emotionality

**DOI:** 10.3389/fpsyg.2017.01861

**Published:** 2017-10-20

**Authors:** Alexander Strobel, Kristin Anacker, Anja Strobel

**Affiliations:** ^1^Department of Psychology, Faculty of Science, Technische Universität Dresden, Dresden, Germany; ^2^Department of Psychology, Faculty of Behavioral and Social Sciences, Chemnitz University of Technology, Chemnitz, Germany

**Keywords:** need for cognition, life events, positive emotionality, negative emotionality, resilience

## Abstract

Need for Cognition (NFC) is conceptualized as an individuals’ tendency to engage in and enjoy effortful cognitive activity and, thus, captures one’s cognitive engagement. It plays a well-established role in information processing in experimental or academic contexts. However, so far comparably little is known about its consequences for other than purely cognitive or academic outcomes. Indeed, NFC is positively associated with personality traits pertaining to Positive Emotionality (PE) and negatively to traits related to Negative Emotionality (NE). Moreover, evidence suggests NFC to be related to an active, problem-focused coping style. We therefore hypothesized NFC to mediate between life events and individual differences in PE and NE. In a sample of *N* = 202 volunteers from the general population, we observed that the number of past positive and negative life events had direct effects on PE, and NE, respectively, and that for positive life events, a mediating effect on PE via NFC was observed, with a higher number of past positive life events being related to higher NFC that in turn was related to increased PE. Thus, the present results lend support to the notion of NFC as an important factor supporting personal well-being by way of its mediating role between the number of past positive life events and positive affect.

## Introduction

Need for Cognition (NFC) is conceptualized as an individual’s tendency to engage in and enjoy effortful cognitive activity. Individuals high in NFC are assumed to rather engage in central, elaborated information processing and those low in NFC to rather engage in peripheral, less elaborated processing (Cacioppo and Petty, 1982; Cacioppo et al., 1996). Ample evidence suggests NFC to be a useful predictor of individual differences in information processing, problem solving, and decision-making (for reviews, see Cacioppo et al., 1996; Petty et al., 2009). Accordingly, NFC has been demonstrated to be related to ability and personality traits that foster effective and efficient information processing: it is positively associated with both fluid and crystallized intelligence and with personality traits related to openness to experience, positive affect, conscientiousness, drive, and achievement-striving (Cacioppo et al., 1996; Fleischhauer et al., 2010; Hill et al., 2013; von Stumm and Ackerman, 2013). Thus, NFC can be considered as a useful construct in research on individual differences in information processing. This also becomes apparent when looking at the relation of NFC with better performance in different types of academic tasks (e.g., Dornic et al., 1991; Kardash and Noel, 2000) and at its positive relation to academic performance in school and university (Richardson et al., 2012; von Stumm and Ackerman, 2013; Luong et al., 2017).

However, apart from its well-confirmed role in information processing in experimental or academic contexts, comparably little is known about consequences of high NFC for other outcomes than purely cognitive or academic ones. The mentioned association of NFC with positive affect together with reportedly lower scores in negative affect (Fleischhauer et al., 2010) as well as in anxiety and depression (Reeves et al., 1995; Epstein et al., 1996; Bertrams and Dickhäuser, 2012) may suggest that, beyond showing better performance in academic settings and cognitively demanding tasks, individuals high in NFC may also feel better while doing so. Indeed, this notion is supported by studies showing that among college students, NFC has beneficial effects on study satisfaction (Grass et al., 2017) and life satisfaction (Coutinho and Woolery, 2004; Gauthier et al., 2006).

It can be assumed that – especially cognitively – challenging life situations should favor high NFC, and that conversely, NFC should develop via observing and experiencing success with a problem-solving-focused style of coping with the challenges of life (cf. Cacioppo et al., 1996). NFC is conceptually and empirically related to Openness to Experience, Extraversion, and Conscientiousness (Fleischhauer et al., 2010) that have been demonstrated to be associated with perceiving life events rather as challenges, with positive appraisals of life situations, and with the employment of problem-focused and engagement-related coping strategies (Penley and Tomaka, 2002; Connor-Smith and Flachsbart, 2007; Carver and Connor-Smith, 2010). By inference, NFC should also be related to such coping styles. Indeed, supporting evidence for this notion comes from research in the context of Cognitive-Experiential Self-Theory by Epstein (1973). In this theory, an intuitive-experiential and an analytical-rational thinking style are contrasted, with an adaptation of the NFC scale (Cacioppo and Petty, 1982) being used to assess individual differences in the latter. Evidence from this line of research suggests that individuals high in NFC are more constructive in thinking, avoid negative overgeneralization as well as unrealistic optimism, and take effective action to solve problems (Epstein et al., 1996). This evidence on NFC-specific coping styles when facing life challenges is underscored by the results of a more recent study on the role of NFC in coping with the transition to retirement: Bye and Pushkar (2009) demonstrated that higher NFC predicted higher positive affect, an effect that was mediated by problem-focused coping and the frequency of cognitive activity.

With the present research, we followed up on this result using a dataset obtained within the context of a larger project on molecular genetic and environmental influences on personality traits (Anacker et al., 2013). We examined this dataset with respect to the role of positive and negative life events in the modulation of traits pertaining to positive and negative affect, or emotionality, being defined by higher-order traits such as Extraversion and Neuroticism (Costa and McCrae, 1992), and Positive and Negative Affect (Watson et al., 1988). Recent evidence has shown that personality traits can be both the antecedent and the consequence of the experience of major life events (e.g., Scollon and Diener, 2006; Specht et al., 2011). Here, we adopted the latter perspective that had also been taken by Bye and Pushkar (2009) and examined the impact of positive and negative life events on positive emotionality (PE) and negative emotionality (NE) and a possible mediation via NFC. Based on the previous evidence outlined above, we hypothesized NFC to mediate between life events and individual differences in traits pertaining to PE and NE. To this end, we examined the impact of the number of self-reported positive and negative life events encountered so far on PE and NE both directly and via possible mediating effects of NFC.

## Materials and Methods

### Participants

The sample consisted of *N* = 202 volunteers (46% men, mean age ±*SD*: 36.8 ± 11.4 years, range: 18–60 years), who had been recruited from the local general population within the context of a larger project on molecular genetic and environmental influences on personality. Based on this project, we already published results (Anacker et al., 2013), however, on an entirely different research topic. A majority of participants (69%) held a university entrance diploma, the remaining 31% held a certificate of secondary education, i.e., had graduated after 10 years of schooling. The sample comprised only participants that had not been diagnosed before for any psychiatric disorder as assessed using the M.I.N.I. International Neuropsychiatric Interview (Sheehan et al., 1998) in its German translation (Ackenheil et al., 1999), given the likely impact of past psychiatric diagnoses on present self-reports of individual levels of PE and NE.

### Procedure

A sample of residents (*N* = 6.775) of the city of Dresden was randomly drawn from the local residents’ registration office. They received a letter with the study description and an invitation to take part in our study. Interested individuals were screened via telephone interviews for a number of inclusion criteria (among them age range 18–60 years, middle European ancestry, German as mother tongue, not taking medication likely to impact on the central nervous system, modest consumption of legal or illegal drugs, and not having received psychotherapy or having been diagnosed for relevant neuropsychiatric conditions within the past 3 years). Upon inclusion, they were invited for personality and molecular genetic assessment, the latter being not of interest here, leaving *N* = 524 participants. After giving informed consent ahead of the assessment, they filled in several biographical assessment sheets as well as personality questionnaires, of which those related to the broad personality traits positive and NE and to NFC were used in the present study (see section Measures). Other questionnaires related more specifically to anxiety and depression were not used for the sake of succinctness and the balance of traits pertaining to PE and NE. A few months later, all participants were again invited to fill in another online questionnaire that was designed to assess both positive and negative life events (see section Measures). The online assessment was done using LimeSurvey (LimeSurvey Project Team and Schmitz, 2015). Participants had the opportunity to win 20 EUR out of a pool of 50 × 20 EUR upon leaving their email addresses that were strictly kept apart from the questionnaire data in a separate LimeSurvey database. 252 participants responded to our call, and after exclusion of those participants with a history of psychiatric disorders (which we considered important as psychiatric conditions may have been coinciding with, resulting from or leading to critical life events), the final sample of *N* = 202 remained for the present analyses. When comparing them with the 322 participants of the larger dataset of *N* = 524 participants, significantly higher NFC was found in the present sample (*t*_454.8_ = -3.88, *p* < 0.001), but no significant differences in variances were present (*F*_321,201_ = 1.1923, *p* = 0.173). Also, the present sample had lower NEO Neuroticism scores (*t*_467.9_ = 4.41, *p* < 0.001) and exhibited a reduced variance (*F*_321,201_ = 1.30, *p* = 0.042), pointing to lower NE with a restriction of range in the present sample. No differences emerged for NEO Extraversion, neither for the mean scores (*t*_425.2_ = 0.87, *p* = 0.384) nor the variances (*F*_321,201_ = 0.99, *p* = 0.920). No differences in age and sex composition were observed (*p* ≥ 0.441), but the participants of the present sample were more likely to have a university entrance diploma (χ^2^ = 13.83, *df* = 1, *p* < 0.001). Thus, the present sample differed in some key variables from the larger sample: it comprised more well-educated and cognitively interested participants with lower NE levels and a restricted NE range (see section Limitations).

The study was carried out in accordance with the recommendations of the German Psychological Society (DGPs). All subjects gave written informed consent in accordance with the Declaration of Helsinki. The protocol was approved by the ethics committee of the DGPs (reference number: ASKPLBB21092007DGPS).

### Measures

As core constituent variables of the broader constructs NE and PE, we used the following measures: the Neuroticism and Extraversion scores of the Revised NEO-Personality Inventory (NEO-PI-R; Costa and McCrae, 1992) in its German translation (Ostendorf and Angleitner, 2004) and the Trait Negative and Positive Affect scores of the Positive and Negative Affect Schedule (PANAS; Watson et al., 1988) in its German version (Krohne et al., 1996), with all scales showing high internal consistency (Cronbach’s α > 0.80). NFC was assessed using the German 16-item version of the NFC scale (Bless et al., 1994) that also has high internal consistency of Cronbach’s α > 0.80 (Bless et al., 1994; Fleischhauer et al., 2010).

The number and evaluation of positive and negative life events was measured using a 79-item self-report questionnaire based on the Munich Event List (Wittchen et al., 1989). Personal life events were assessed across 12 domains (life events related to parents/carers, siblings, partners, children, friends, significant others, health and trauma, education and work, leisure, living, finances, and property issues, and legal and political/societal issues). Participants indicated how often (on a 5-point rating scale ranging from 0 to 4 and more), when and for how long they had experienced each of these events, and how they evaluated these experiences on a 7-point rating scale ranging from -3 = very negative to +3 = very positive. Crucially, in this context, participants were also asked to consider the positive impact of objectively negative life events and vice versa. In the present study, we used the number of positive life events (nPLE: *M* = 27.7, *SD* = 9.9, range = 6–68) and negative life events (nNLE: *M* = 12.9, *SD* = 8.6, range: 0–51) encountered so far. We also calculated the sums of their respective evaluation (ePLE: *M* = 53.4, *SD* = 24.9, range = 3–154; and eNLE: *M* = -26.4, *SD* = 20.0, range = -131–0), but as number and evaluation scores were highly or nearly perfectly correlated (PLE: *r* = 0.86, NLE: *r* = -0.97, both *p* < 0.001), the statistical analyses were based solely on the number of positive and negative life events.

### Statistical Analyses

**FIGURE 1:**
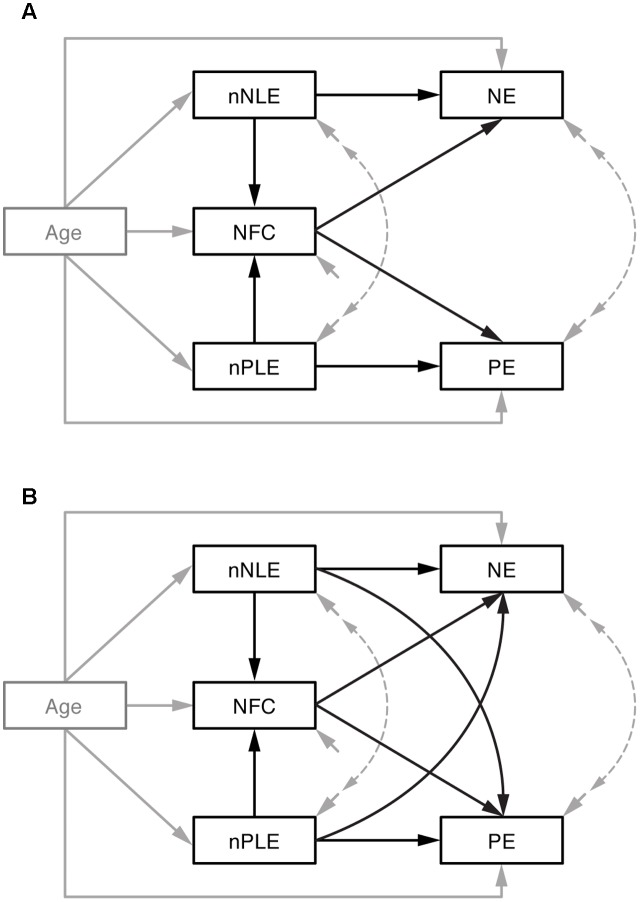
Mediation models. **(A)** Preferred model of the relationship between the number of positive and negative life events (nPLE and nNLE) on Negative Emotionality (NE) and Positive Emotionality (PE), both directly and via Need for Cognition (NFC), with all variables regressed on Age. Covariate effects, error variances and their correlations (dashed lines) are given in gray, the mediation model is given in black. **(B)** Alternative model with additional paths from nNLE to PE and nPLE to NE.

All analyses were performed using RStudio (RStudio Team, 2015) with R 3.3.1 (R Core Team, 2016) using the additional packages *psych* (Revelle, 2016), *car* (Fox and Weisberg, 2011), and *lavaan* (Rosseel, 2012). As almost all of the variables of interest deviated from univariate normality (Shapiro–Wilk tests, *p* between 0.159 and 0.001), the variables were normalized using Blom’s formula [(*r* - 3/8)/(*n* + 1/4), with *r* being the rank of observations and *n* the sample size; Blom, 1958], with their original means and standard deviations being retained. The normalized variables did not deviate from univariate normality (Shapiro–Wilk tests, *p* ≥ 0.894) with the exception of the normalized PANAS-NA scores that still showed some, albeit acceptable deviation from normality (*p* = 0.098). Afterwards, all variables including the *a priori* covariates age, sex, and educational level, operationalized by holding or not holding a university entrance diploma, were mean-centered. Then, intercorrelations of the variables of interest were calculated as Pearson correlations with Holm-adjustment for multiple testing as implemented in the *psych* package. Then, again using *psych*, an exploratory principal axis factor analysis with Varimax rotation was performed with standardized NEO-N, NEO-E, PANAS-NA, and PANAS-PA as input variables, and regression-based scores for the resulting factors PE and NE were extracted (see section Results). We chose orthogonal rotation to yield uncorrelated scores for the sake of easier interpretability of the subsequent mediation analyses, i.e., possible effects for PE being independent of NE and *vice versa*. The main analyses were performed using the *lavaan* package: a mediation model (**Figure 1**) was defined with nPLE and nNLE as independent variables, NFC as mediator, and the NE and PE factor scores as dependent variables. All variables in the model were regressed on age to control for possible confounding effects of this covariate as age can be assumed to be positively related to the number of life events (see also **Table 1**). We assumed direct paths from nNLE to NE and nPLE to PE (**Figure 1A**). We further assumed paths from nNLE and nPLE to NFC, with the latter being more likely, given that positive life events should have a stronger impact on NFC than negative ones. Finally, we assumed paths from NFC to NE and PE and calculated indirect effects of nNLE and nPLE on NE and PE via NFC. Additionally, given the correlation between nNLE and nPLE (**Table 1**), we assumed correlated error variances of these variables. As Mardia tests of multivariate skew and kurtosis indicated no deviation from multivariate normality (all *p* ≥ 0.65), the path model was estimated via maximum likelihood estimation. Bootstrapped standard errors and probabilities of test statistics were determined based on 1000 bootstrap samples. We also fitted two alternative models: one without the mediator paths and one with additional paths from nNLE to PE and from nPLE to NE (**Figure 1B**). These models were compared to the preferred model via χ^2^ difference tests. Two final analyses were performed: (1) To control for a possible influence of educational level that can be considered to be associated with NFC (see also **Table 1**), we estimated a model that controlled for this influence. (2) To examine possible differences between analyses based on normalized scores and raw scores, the preferred model was also estimated using the raw scores.

**Table 1:** Intercorrelations of the variables and covariates of interest.

		1	2	3	4	5	6	7	8	9	10	11
1	Age	–										
2	Sex	-0.03	–									
3	Edu	-**0.24**	0.08	–								
4	nNLE	**0.30**	0.05	-0.15	–							
5	nPLE	**0.52**	-0.05	0.01	**0.29**	–						
6	NFC	0.10	-0.20	**0.27**	0.05	**0.31**	–					
7	NEO-N	-**0.23**	0.16	-0.09	0.22	-**0.31**	-**0.31**	–				
8	NEO-E	-0.17	0.13	0.10	-0.02	0.17	**0.26**	-**0.35**	–			
9	PANAS-NA	-0.11	0.04	0.01	0.15	-0.12	-0.08	**0.63**	-**0.28**	–		
10	PANAS-PA	0.07	0.20	0.08	0.00	**0.31**	**0.34**	-**0.36**	**0.61**	-0.16	–	
11	NE	-0.14	0.10	-0.01	0.19	-0.15	-0.11	*0.68*	-*0.25*	*0.88*	-*0.10*	–
12	PE	0.03	0.20	0.09	0.01	**0.30**	**0.35**	-*0.30*	*0.63*	-*0.08*	*0.93*	-0.05


*Edu, educational level (absence vs. presence of university entrance diploma); nNLE, number of negative life events; nPLE, number of positive life events; NFC, Need for Cognition; NEO-N, NEO-PI-R Neuroticism; NEO-E, NEO-PI-R Extraversion; PANAS-NA, PANAS Traits Negative Affect; PANAS-PA, PANAS Traits Positive Affect; Coefficients are Pearson correlations, bold-faced coefficients *P* < 0.05 (after Holm-adjustment for multiple testing), except for coefficients in italics that give the factor loadings of the constituent variables on the factor-analytically derived and Varimax-rotated factors. NE, Negative Emotionality and PE, Positive Emotionality, with the last entry (# 11 × 12) giving the remaining intercorrelation of the NE and PE factor scores*.

## Results

### Correlational Analyses

**Table 1** gives the correlations of all variables examined here, together with their significance levels (please note: *p*-values are Holm-adjusted for multiple testing and thus can be 1). nPLE and nNLE were correlated (*r* = 0.29, *p* = 0.001), but only nPLE showed a significant association with NFC (*r* = 0.31, *p* < 0.001). The variables assumed to pertain to the factor NE (i.e., NEO-N and PANAS-NA) were highly intercorrelated, as were those related to the factor PE (i.e., NEO-E and PANAS-PA). Principal axis factor analysis of the variables NEO-N and -E, and PANAS-NA, and -PA, followed by Varimax rotation yielded the two factors PE and NE (Eigenvalues 2.20 and 1.06, explained variance 34 and 33%). The NE factor scores showed no significant relation to nNLE (*r* = 0.19, *p* = 0.288) and did not significantly correlate with NFC (*r* = -0.11, *p* = 1). The PE factor scores were significantly correlated with both nPLE (*r* = 0.30) and NFC (*r* = 0.35, both *p* < 0.001).

### Impact of the Number of Negative and Positive Life Events on NE and PE

**FIGURE 2:**
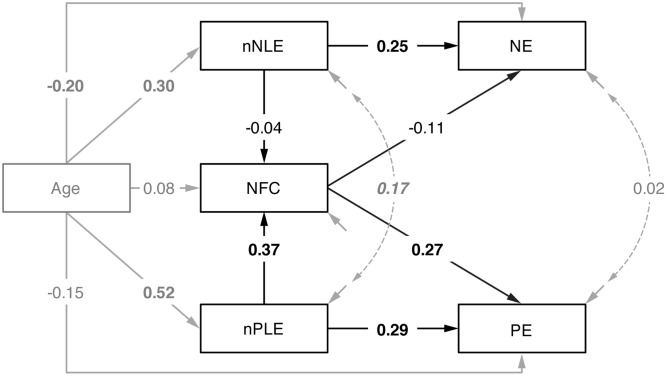
Mediation results. Influence (β estimates, bold-faced: *p* ≤ 0.01, italics: *p* < 0.05) of nPLE and nNLE on NE and PE, both directly and via NFC, with all variables regressed on Age. Covariate effects, error variances (coefficients omitted for clarity) and their correlations (dashed lines) are given in gray, the mediation model is given in black. nNLE directly impacts on NE without a mediating role of NFC (β < 0.01, *p* = 0.654), while for nPLE, the indirect effect in PE via NFC is significant (β = 0.10, *p* = 0.014).

Fit of the mediation model (**Figure 2**) was excellent [χ^2^ = 3.01, *df* = 2, *p* = 0.222, CFI = 0.99, RMSEA = 0.05 (90% confidence interval: 0.00, 0.16), SRMR = 0.02]. Age had a significant influence on both nNLE and nPLE (with higher age being related to a higher number of reported significant life events) and also on NE (with higher age being related to lower NE levels). nNLE directly impacted on NE (β = 0.25, *p* = 0.002) without an indirect effect via NFC (β < 0.01, *p* = 0.654). For nPLE, in addition to the direct effect on PE (β = 0.29, *p* = 0.001), the indirect effect on PE via NFC was significant (β = 0.10, *p* = 0.014), suggesting that NFC acted as partial mediator of the role of the number of positive life events on PE. The total effect of the number of life events and NFC (i.e., direct + indirect effects) was significant for both NE (β = 0.21, *p* = 0.010) and PE (β = 0.38, *p* < 0.001). The total model’s explained variance was *R*^2^ = 0.09 for NE and *R*^2^ = 0.18 for PE.

We also compared our model to two alternative models: In a model without the mediator paths, the direct path from nNLE to NE remained the same (β = 0.25, *p* = 0.002), while that from nPLE to PE was higher than in the model above (β = 0.39, *p* < 0.001). However, this model had a bad fit [χ^2^ = 41.34, *df* = 6, *p* < 0.001, CFI = 0.78, RMSEA = 0.17 (90% confidence interval: 0.12, 0.22), SRMR = 0.10] and was significantly inferior to the preferred model (χ_diff_^2^ = 38.33, *df*_diff_ = 4, *p* < 0.001), indicating that inclusion of the mediator paths considerably improved fit. Moreover, the explained variance was considerably smaller for PE (*R*^2^ = 0.11; for NE: *R*^2^ = 0.08). A model with paths also from nNLE to PE and from nPLE to NE did not significantly differ from the former model (χ_diff_^2^ = 3.01, *df*_diff_ = 2, *p* = 0.222), suggesting that these (actually insignificant) paths did not improve the model. Finally, given the correlation of educational level and NFC, we also performed an additional analysis controlling for this influence, and because we relied on normalized scores, we also estimated a model using raw scores. In both analyses, the results remained essentially unchanged.

## Discussion

In the present study, we found that the number of past positive and negative life events had direct effects on PE, and NE, respectively. Moreover, for PE, a prominent mediating effect of NFC was observed: a higher number of past life events was related to higher NFC that in turn was associated with increased PE. Thus, the present results lend support to the notion of NFC as an important factor supporting personal well-being by way of its mediating role between the number of past positive life events and positive affect. Note that the mediation model also considered influences of age, hence, the present results are not simply attributable to a higher number of life events with increasing age coinciding with age-related changes in PE and NE just by chance.

### Impact of the Number of Positive and Negative Life Events on PE and NE

Our results argue for a valence-specific role of past life events in molding emotionality. There were significant, albeit small to medium, direct effects of past *negative* life events on NE, and of past *positive* life events on PE. They are in line with evidence pointing to personality changes being at least partly attributable to individual experiences (e.g., Scollon and Diener, 2006; Specht et al., 2011). Most likely, this is due to correlated change, i.e., personality traits shape one’s experiences that in turn modulate trait levels and so on. Having cross-sectional data, we could not address this issue. However, the main focus of the present research was on a possible mediating role of NFC. Indeed, the effect of positive life events on PE was significantly mediated by NFC, with a more positive evaluation of past life events being positively associated with NFC, that in turn positively impacted on PE. While one has to bear in mind that this mediation effect was small, the explained variance in PE was substantially larger when NFC was included as mediator. These results suggest that positive life events may not only have a directly enhancing effect on PE, but that their appraisal as events conducive of personal growth may also indirectly enhance PE and, thus, by inference, personal well-being and mental health. Moreover, generalizing animal research on beneficial effects of positive environmental enrichment on cognitive functioning and well-being (Young et al., 1999; Brydges et al., 2011), it can be assumed that a diversity of positive life events enriches an individual’s experience. Via learning, this adds to the successful accommodation to future life events. It can further be assumed that this should be the case especially in individuals who are habitually oriented toward a problem-solving oriented style of coping with life’s challenges. Such a coping style may, in theory, develop based on experiences of mastery of life challenges via expenditure of cognitive effort (Cacioppo et al., 1996). Indeed, it appears to be pertinent to NFC, both by inference from similar traits (e.g., Connor-Smith and Flachsbart, 2007; Carver and Connor-Smith, 2010) and by reference to more direct evidence (Epstein et al., 1996).

In this context, one issue deserves special mention: The line of arguments above bear some similarity with the reasoning behind the *behavioral activation* aspect of Cognitive-Behavioral Therapy of depression. This aspect of the therapy is explicitly oriented toward raising the number of life events, with the focus on raising reinforcing life events (Kanter et al., 2010). In addition to its expected direct impact on increasing PE, the present results may point to an additional indirect influence via NFC. Further research should thus explore the possibility that individuals with higher NFC levels would benefit more from *behavioral activation* than those with lower NFC.

As mentioned, an alternative model positing NFC as mediator between positive and NE as antecedents and the experience of positive and negative life events as outcomes would also be a possibility, but would still underscore NFC’s mediating role. Moreover, an explanation of the effects observed here could also be based on the assumption of personality-dependent memory biases. One could assume that there is an interaction between a better recall and recognition performance of individuals high in NFC (e.g., Kardash and Noel, 2000), their elaborated information processing focusing on more relevant aspects of a situation (Cacioppo and Petty, 1982; Cacioppo et al., 1996), and their experience to be able to deal with challenging life situations. Having a broader basis of situations to refer to especially with a focus on the aspects of these situations that helped to cope with them could in turn support the development of more effective coping strategies for future situations. This could also result in a more positive evaluation of challenging life events. These issues could not be addressed with the current data and research design, future studies should therefore use longitudinal designs. Moreover, they should also take measures to exclude that mediating effects of NFC on affective outcomes are only due to personality-dependent memory biases, e.g., via objective life data, informant reports, or experience sampling procedures.

### Limitations

A first limitation regards the fact that while our study was motivated by the findings of Bye and Pushkar (2009), we could not attempt to directly replicate their work, but rather had to rely on data that had been collected not exactly for that purpose. Hence, our results can only be seen as conceptual replication. Nevertheless, our work and that of Bye and Pushkar (2009) converge in the conclusion that NFC appears to be an important mediator of the impact of life events on positive affect.

Another issue that needs to be discussed is the problem of multiple testing. It has been argued that the statistical significance of multiple parameters in a structural equation model should be controlled for with regard to Type I errors, e.g., via False Discover Rate (FDR) control (Cribbie, 2007). Indeed, in the preferred model, we estimated 24 parameters (eleven paths, five error variances, two correlations between error variances, four indirect and two total effects). Employing FDR control, all of our nominally significant parameters remained significant with the highest *p*-value below 0.05 being *p*_uncorr_ = 0.017 and *p*_FDR_ = 0.025. Thus, we consider our results as not simply due to multiple testing.

A further issue regards power. Our sample size of *N* = 202 enabled us to detect even small correlations of 0.19 at an α-level of 0.05 and a power of 1–β of 0.80 as determined using G^∗^Power 3.1 (Faul et al., 2009). Thus, we consider the power of our sample as adequate, the more so as *N* > 200 is often recommended for structural equation modeling. Nevertheless, Schönbrodt and Perugini (2013) showed via simulations that for correlations, stable estimates can be expected only in samples larger than about *N* = 250. It is conceivable that the parameter in question here, i.e., the indirect effect of nPLE on PE via NFC, did not reach stability in *N* = 202. We therefore performed a similar analysis as done by Schönbrodt and Perugini (2013) and estimated the preferred model successively in samples with increasing size, starting from the first ten participants. The parameter estimate for the indirect effect was extracted and plotted together with the corridor defined by the bootstrapped 95% confidence interval of this indirect effect in the full sample. The parameter estimate did not leave this corridor anymore for *N* > 32, arguing for the stability of our estimate.

A third point regards the sample composition. While we had drawn our sample from the local general population based on the local register of residents, we still had to rely on voluntary participation. This, however, would in principle result in rather lower-bound estimates of the effects observed here due to a restriction in range and not in inflated ones. Thus, our sample may rather have led to obscured than to exaggerated effects. However, compared to those who initially also took part in the larger project, but were excluded because of past psychiatric diagnoses or did not respond to our call for another assessment at all, the present sample differed in some key variables from the larger sample: it was composed of more well-educated and cognitively interested participants with lower levels of NE and a restricted range in NE (see section Materials and Methods for details). The latter issue may have obscured effects with regard to NE. The former issue reduces the generalization of our results and calls for independent replication.

## Conclusion and Future Directions

We are not the first to propose a positive impact of NFC on PE in view of challenging life situations. As mentioned, NFC has been found to predict coping ability (Epstein et al., 1996), life and study satisfaction in students (Coutinho and Woolery, 2004; Grass et al., 2017), and positive affect during the transition to retirement (Bye and Pushkar, 2009). Hence, our data add to the emerging evidence on a positive role of NFC in mastering life challenges by suggesting NFC as a mediator of the relationship between positive life events and PE as one factor implicated in the resilience to psychopathological outcomes (Watson et al., 2015). Future research on that topic should employ longitudinal designs to examine more closely the mediating role of NFC in a most likely correlated change of positive life events and PE.

## Author Contributions

AlS and KA conceived and designed the study, KA performed data collection and pre-processing, AlS analyzed the data with the help of AnS, AlS and AnS wrote, and KA critically commented on the manuscript, AlS, AnS, and KA gave final approval of the manuscript to be published.

## Conflict of Interest Statement

The authors declare that the research was conducted in the absence of any commercial or financial relationships that could be construed as a potential conflict of interest.

## References

[B1] Ackenheil M. Stotz-Ingenlath G. Dietz-Bauer R. Vossen A. (1999). *M.I.N.I. Mini International Neuropsychiatric Interview, German Version 5.0.0.* München: Psychiatrische Universitätsklinik München.

[B2] Anacker K. Enge S. Reif A. Lesch K. P. Strobel A. (2013). Dopamine D4 receptor gene variation impacts self-reported altruism. *Mol. Psychiatry* 18 402–403. 10.1038/mp.2012.49 22565783

[B3] Bertrams A. Dickhäuser O. (2012). Passionate thinkers feel better: self-control capacity as mediator of the relationship between need for cognition and affective adjustment. *J. Individ. Dif.* 33 69–75. 10.1027/1614-0001/a000081

[B4] Bless H. Wänke M. Bohner G. Fellhauer R. L. Schwarz N. (1994). Need for cognition: eine skala zur erfassung von engagement und freude bei denkaufgaben [Need for cognition: a scale measuring engagement and happiness in cognitive tasks]. *Z. Sozialpsychol.* 25 147–154.

[B5] Blom G. (1958). *Statistical Estimates and Transformed Beta Variables.* New York, NY: John Wiley.

[B6] Brydges N. M. Leach M. Nicol K. Wright R. Bateson M. (2011). Environmental enrichment induces optimistic cognitive bias in rats. *Anim. Behav.* 81 169–175. 10.1016/j.anbehav.2010.09.030

[B7] Bye D. Pushkar D. (2009). How need for cognition and perceived control are differentially linked to emotional outcomes in the transition to retirement. *Motiv. Emot.* 33 320–332. 10.1007/s11031-009-9135-3

[B8] Cacioppo J. T. Petty R. E. (1982). The need for cognition. *J. Pers. Soc. Psychol.* 42 116–131. 10.1037//0022-3514.42.1.116

[B9] Cacioppo J. T. Petty R. E. Feinstein J. A. Jarvis W. B. G. (1996). Dispositional differences in cognitive motivation. *Psychol. Bull.* 119 197–253. 10.1037/0033-2909.119.2.197

[B10] Carver C. S. Connor-Smith J. (2010). Personality and coping. *Annu. Rev. Psychol.* 61 679–704. 10.1146/annurev.psych.093008.100352 19572784

[B11] Connor-Smith J. K. Flachsbart C. (2007). Relations between personality and coping: a meta-analysis. *J. Pers. Soc. Psychol.* 93 1080–1107. 10.1037/0022-3514.93.6.1080 18072856

[B12] Costa P. T. McCrae R. R. (1992). *NEO PI-R Professional Manual.* Odessa, FL: Psychological Assessment Resources, Inc.

[B13] Coutinho S. A. Woolery L. A. (2004). The need for cognition and life satisfaction among college students. *Coll. Stud. J.* 38 203–206.

[B14] Cribbie R. A. (2007). Multiplicity control in structural equation modeling. *Struct. Equ. Model. Multidiscip. J.* 14 98–112. 10.1080/10705510709336738

[B15] Dornic S. Ekehammar B. Laaksonen T. (1991). Tolerance for mental effort - self-ratings related to perception, performance and personality. *Pers. Individ. Dif.* 12 313–319. 10.1016/0191-8869(91)90118-U

[B16] Epstein S. (1973). Self-concept revisited or a theory of a theory. *Am. Psychol.* 28 404–416. 10.1037/h0034679 4703058

[B17] Epstein S. Pacini R. DenesRaj V. Heier H. (1996). Individual differences in intuitive-experiential and analytical-rational thinking styles. *J. Pers. Soc. Psychol.* 71 390–405. 10.1037/0022-3514.71.2.390 8765488

[B18] Faul F. Erdfelder E. Buchner A. Lang A.-G. (2009). Statistical power analyses using G^∗^Power 3.1: tests for correlation and regression analyses. *Behav. Res. Methods* 41 1149–1160. 10.3758/BRM.41.4.1149 19897823

[B19] Fleischhauer M. Enge S. Brocke B. Ullrich J. Strobel A. Strobel A. (2010). Same or different? Clarifying the relationship of need for cognition to personality and intelligence. *Pers. Soc. Psychol. Bull.* 36 82–96. 10.1177/0146167209351886 19901274

[B20] Fox J. Weisberg S. (2011). *An R Companion to Applied Regression.* Thousand Oaks, CA: Sage.

[B21] Gauthier K. J. Christopher A. N. Walter M. I. Mourad R. Marek P. (2006). Religiosity, religious doubt, and the need for cognition: their interactive relationship with life satisfaction. *J. Happiness Stud.* 7 139–154. 10.1007/s10902-005-1916-0

[B22] Grass J. Strobel A. Strobel A. (2017). Cognitive investments in academic success: the role of need for cognition at university. *Front. Psychol.* 8:790. 10.3389/fpsyg.2017.00790 28559876 PMC5432647

[B23] Hill B. D. Foster J. D. Elliott E. M. Shelton J. T. McCain J. Gouvier W. D. (2013). Need for cognition is related to higher general intelligence, fluid intelligence, and crystallized intelligence, but not working memory. *J. Res. Pers.* 47 22–25. 10.1016/j.jrp.2012.11.001

[B24] Kanter J. W. Manos R. C. Bowe W. M. Baruch D. E. Busch A. M. Rusch L. C. (2010). What is behavioral activation? A review of the empirical literature. *Clin. Psychol. Rev.* 30 608–620. 10.1016/j.cpr.2010.04.001 20677369

[B25] Kardash C. M. Noel L. K. (2000). How organizational signals, need for cognition, and verbal ability affect text recall and recognition. *Contemp. Educ. Psychol.* 25 317–331. 10.1006/ceps.1999.1011 10873375

[B26] Krohne H. W. Egloff B. Kohlmann C. W. Tausch A. (1996). Investigations with a German version of the positive and negative affect schedule (PANAS). *Diagnostica* 42 139–156.

[B27] LimeSurvey Project Team and Schmitz C. (2015). *LimeSurvey: An Open Source Survey Tool.* Hamburg: LimeSurvey Project.

[B28] Luong C. Strobel A. Wollschläger R. Greiff S. Vainikainen M.-P. Preckel F. (2017). Need for cognition in children and adolescents: behavioral correlates and relations to academic performance and academic potential. *Learn. Individ. Differ.* 53 103–113. 10.1016/j.lindif.2016.10.019

[B29] Ostendorf F. Angleitner A. (2004). *NEO-PI-R - NEO Persönlichkeitsinventar nach Costa und McCrae - Revidierte Fassung.* Göttingen: Hogrefe.

[B30] Penley J. A. Tomaka J. (2002). Associations among the Big Five, emotional responses, and coping with acute stress. *Pers. Individ. Dif.* 32 1215–1228. 10.1016/S0191-8869(01)00087-3

[B31] Petty R. E. Brinþol P. Loersch C. McCaslin M. J. (2009). “The need for cognition,” in *Handbook of Individual Differences in Social Behavior* eds Leary M. R. Hoyle R. H. (New York, NY: Guilford Press) 318–329.

[B32] R Core Team (2016). *R: A Language and Environment for Statistical Computing.* Vienna: R Foundation for Statistical Computing.

[B33] Reeves A. L. Watson P. J. Ramsey A. Morris R. J. (1995). Private self-consciousness factors, Need for cognition, and depression. *J. Soc. Behav. Pers.* 10 431–443.

[B34] Revelle W. (2016). *psych: Procedures for Psychological, Psychometric, and Personality Research.* Evanston, IL: Northwestern University.

[B35] Richardson M. Abraham C. Bond R. (2012). Psychological correlates of university students’ academic performance: a systematic review and meta-analysis. *Psychol. Bull.* 138 353–387. 10.1037/a0026838 22352812

[B36] Rosseel Y. (2012). lavaan: an R package for structural equation modeling. *J. Stat. Softw.* 48 1–36. 10.3389/fpsyg.2014.01521 25601849 PMC4283449

[B37] RStudio Team (2015). *RStudio: Integrated Development Environment for R.* Boston, MA: RStudio Inc.

[B38] Schönbrodt F. D. Perugini M. (2013). At what sample size do correlations stabilize? *J. Res. Pers.* 47 609–612. 10.1016/j.jrp.2013.05.009

[B39] Scollon C. N. Diener E. (2006). Love, work, and changes in extraversion and neuroticism over time. *J. Pers. Soc. Psychol.* 91 1152–1165. 10.1037/0022.3514.91.6.1152 17144771

[B40] Sheehan D. V. Lecrubier Y. Sheehan K. H. Amorim P. Janavs J. Weiller E. (1998). The mini-international neuropsychiatric interview (M.I.N.I.): the development and validation of a structured diagnostic psychiatric interview for DSM-IV and ICD-10. *J. Clin. Psychiatry* 59(Suppl. 20), 22–33. 9881538

[B41] Specht J. Egloff B. Schmukle S. C. (2011). Stability and change of personality across the life course: the impact of age and major life events on mean-level and rank-order stability of the Big Five. *J. Pers. Soc. Psychol.* 101 862–882. 10.1037/a0024950 21859226

[B42] von Stumm S. Ackerman P. L. (2013). Investment and intellect: a review and meta-analysis. *Psychol. Bull.* 139 841–869. 10.1037/a0030746 23231531

[B43] Watson D. Clark L. A. Tellegen A. (1988). Development and validation of brief measures of positive and negative affect - the PANAS scales. *J. Pers. Soc. Psychol.* 54 1063–1070. 10.1037/0022-3514.54.6.1063 3397865

[B44] Watson D. Stasik S. M. Ellickson-Larew S. Stanton K. (2015). Extraversion and psychopathology: a facet-level analysis. *J. Abnorm. Psychol.* 124 432–446. 10.1037/abn0000051 25751628

[B45] Wittchen H. U. Essau C. A. Hecht H. Teder W. Pfister H. (1989). Reliability of life event assessments - Test retest reliability and fall-off effects of the Munich interview for the assessment of life events and conditions. *J. Affect. Disord.* 16 77–91. 10.1016/0165-0327(89)90059-1 2521655

[B46] Young D. Lawlor P. A. Leone P. Dragunow M. During M. J. (1999). Environmental enrichment inhibits spontaneous apoptosis, prevents seizures and is neuroprotective. *Nat. Med.* 5 448–453. 10.1038/7449 10202938

